# Implementation and Validation of Free Flaps in Acute and Reconstructive Burn Care

**DOI:** 10.3390/medicina57070718

**Published:** 2021-07-16

**Authors:** Benjamin Ziegler, Gabriel Hundeshagen, Jan Warszawski, Emre Gazyakan, Ulrich Kneser, Christoph Hirche

**Affiliations:** 1 Department of Hand, Plastic and Reconstructive Surgery, Microsurgery—Burn Center, BG Trauma Center Ludwigshafen/Rhine, University Heidelberg, Ludwig-Guttmann-Str. 13, 67071 Ludwigshafen, Germany; Benjamin.Ziegler@bgu-frankfurt.de (B.Z.); gabriel.hundeshagen@bgu-ludwigshafen.de (G.H.); jan.warszawski@bgu-frankfurt.de (J.W.); Emre.Gazyakan@bgu-ludwigshafen.de (E.G.); Ulrich.Kneser@bgu-ludwigshafen.de (U.K.); 2 Department of Plastic, Hand and Reconstructive Microsurgery, Hand Trauma and Replantation Center (FESSH), BG Unfallklinik Frankfurt am Main gGmbH, Academic Teaching Hospital of Goethe-University of Frankfurt, Friedberger Landstrasse 430, 60389 Frankfurt am Main, Germany

**Keywords:** burns, microsurgery, reconstruction, free flap

## Abstract

Microsurgical free flap reconstruction in acute burn care offers the option of reconstructing even challenging defects in a single stage procedure. Due to altered rheological and hemodynamic conditions in severely burned patients, it bears the risk of a higher complication rate compared to microsurgical reconstruction in other patients. To avoid failure, appropriate indications for free flap reconstruction should be reviewed thoroughly. Several aspects concerning timing of the procedure, individual flap choice, selection and preparation of the recipient vessels, and perioperative measures must be considered. Respecting these specific conditions, a low complication rate, comparable to those seen in microsurgical reconstruction of other traumatic limb defects, can be observed. Hence, the free flap procedure in acute burn care is a relatively safe and reliable tool in the armamentarium of acute burn surgery. In reconstructive burn care, microsurgical tissue transfer is routinely used to treat scar contractures. Due to the more robust perioperative condition of patients, even lower rates of complication are seen in microsurgical reconstruction.

## 1. Introduction

Soft tissue reconstruction by microsurgical free flap reconstruction is a rarely required, but eminent, surgical tool in acute burn care. Due to substantial differences in indication, technique, and outcome, free flap reconstruction in acute burn care and in burn reconstruction must be considered separately. Nonetheless, free flaps for both indications have been described only a very few years after Cobbett’s historical first microsurgical tissue transfer [1]. For example, in 1975 when Sharzer et al. [2] presented the successful transfer of two free groin flaps for acute burn wound reconstruction, and when Harii et al. [3] reported 47 free flap procedures for secondary reconstruction of 36 contractured or unstable burn scars in the same year.

## 2. Indication

**Figure 1:**
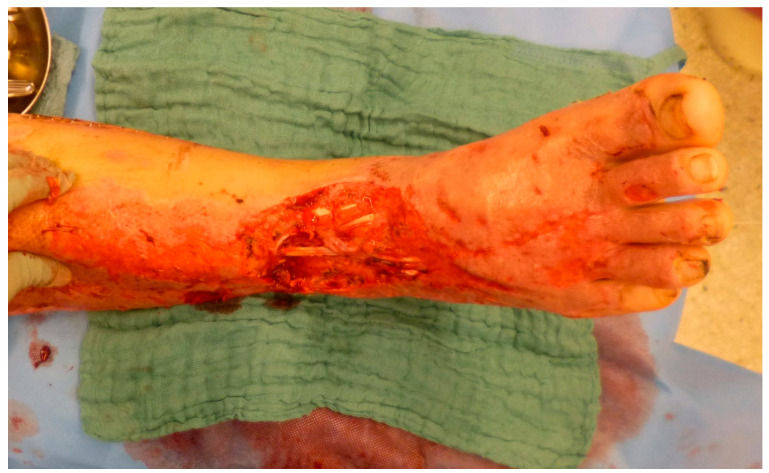
Full-thickness defect after failed STSG due to diminished perfusion of the wound bed in a patient suffering from high-voltage trauma.

Severe burn injuries usually involve loss of skin and soft tissue and often require extensive wound reconstructions. While most burn wounds are sufficiently reconstructed by split-thickness skin grafts (STSG) [4], some burn wounds pose complex defects with deep tissue necrosis and exposed functional structures like bone, tendon, or nerves after complete eschar removal (cp. Figure 1). These deep burns commonly result from a longer exposure time in flame burns or from contact or scald burns due to the conduction of a higher amount of thermal energy. Electrical trauma and chemical burns are less frequent but can lead to even more devastating tissue defects, even affecting muscle compartments and damage to main vessels or nerves [5]. Because of their delicate soft tissue layers with functional structures directly underneath, hands and feet are often affected and may require free flap coverage in the acute setting, but other areas with thin skin coverage overlying bony prominences like elbow, knee or tibia are also at risk.

Several options are available which offer viable soft tissue coverage for reconstruction of these defects. While use of local flaps is often limited due to restricted availability of nearby unaffected fasciocutaneous tissue, distant pedicled flaps like the groin flap or abdominal bridge flaps are frequently used—at least for the reconstruction of defects on the distal forearm and hand [6,7]. These flaps offer a low failure rate, short operating times, and are technically undemanding, but are limited by the mandatory (partly) immobilization of the treated extremity, usually for a minimum of 2–3 weeks. In addition, unburned donor sites must be available, which limits their use in cases with larger surface area burn injuries.

Dermal matrices and negative pressure wound therapy (NPWT) allow successful skin grafting in areas of exposed bone or tendon in select cases [8,9]. However, stable healing cannot be achieved in all patients using these techniques. Although some progress has been made using different templates and matrices [10], these techniques are still dependent on a minimum degree of perfusion of the wound bed, which is not the situation following high voltage electrical trauma, and are limited in the thickness and quality of the soft tissue they provide [11].

On the other hand, primary microsurgical reconstruction of burn wounds with a devitalized wound bed or exposed functional structures offers a great variety of different free flaps to provide custom-made reconstruction of nearly every possible defect site. Due to fast and stable tissue coverage, early functional training can be begun in the first days after surgery, in order to ensure the best possible functional outcome [12]. Hence, free flap reconstruction should be the first choice for early reconstruction of complex and large defects with exposed bone, nerves, or tendons when local flaps are unavailable in the acute phase.

In secondary burn reconstruction the primary goal is the release and excision of contracted burn scars before irreversible, mutilating contractures emerge with permanent arthrogenic limitations. Soft tissue coverage is needed because scar release always leads to soft tissue defects of variable size. Similar to acute burn reconstruction, smaller defects can be closed by local flaps, e.g., the serial z-plasty or advancement flaps. In more extensive defects, distant or free flap reconstruction competes with the use of dermal substitutes or tissue expansion [13,14]. Apart from functional concerns, aesthetic components of successful reconstruction of mutilating burn scars through microsurgical tissue transfer must also be considered [15].

In addition, vascularized composite allotransplantation (VCA) has been described as an alternative for secondary reconstruction in severe facial burns. It offers total face reconstruction, including reconstruction of functions like nose breathing, eating, and expression [16]. As the required lifelong immunosuppression bears a risk of chronic infections and cancer, and only very few burn centers have access to this technique, it is rarely indicated. In the absence of an international consensus, indications to evaluate the option for face transplant may include soft tissue loss of more than 25% of the face, loss of multiple facial units that are hard to reconstruct, loss of multiple facial functions, or otherwise failed attempts for facial reconstructions [17].

## 3. Timing

In primary burn treatment, timing is an important factor to ensure success of the free flap procedure comparable to reconstruction of other traumatic defects [18]. Literature shows a reduced risk of flap failure when free flap reconstruction is performed early after trauma [19]. A certain hypercoagulability seems to arise 48 h after burn trauma [20], suggesting that a free flap procedure presents a lower risk if performed earlier after trauma. Hence, primary free flap reconstruction during the first days after trauma might be beneficial, at least in burn patients with only one affected extremity who are hemodynamically stable. Of note, these cases, more often associated with thermal crush injuries than with actual flame burns, are rare.

Extensive burn injuries, on the other hand, lead to characteristic burn shock due to vascular hyperpermeability and consecutive fluid shifting in the initial phase after trauma. In these cases, fluid resuscitation and sequential debridement with subsequent skin grafting should be completed to achieve a stable circulatory state prior to planning microsurgical tissue transfer. Definite defect closure can be postponed in these cases by conserving exposed bones or tendons using negative pressure wound therapy hydrogel dressings.

## 4. Flap Choice

As in every free flap procedure, several aspects need to be considered to choose the appropriate free flap for effective defect coverage in acute and non-acute burn reconstruction. Apart from defect size, special reconstructive needs, such as a fascial layer to facilitate tendon movement or reconstruction of a blood vessel to improve local circulation, must be taken into account. In addition, many extensively burned patients offer limited donor sites providing fasciocutaneous free flaps with intact skin or an adequate pedicle length to reach undamaged recipient vessels. In patients with unburned thighs, the anterolateral thigh flap (ALT), first described by Song et al. in 1984 [21], offers a huge versatility when designed as a compound flap. While it is capable of closing even extensive defects when accepting skin grafting of its donor site, it can be raised with fascia if needed. In addition, it can be used as a flow-through flap to reconstruct damaged axial arteries of injured limbs [22]. In the absence of suitable cutaneous donor sites, free muscle flaps can be raised even in massively burned patients and in donor sites showing full-thickness burn wounds. Using free rectus abdominis (Pennington, 1980 [23]), vastus lateralis (Ger, 1976 [24]) or latissimus dorsi muscle flaps (Tansini, 1896 [25]) combined with split-thickness skin grafts (STSG), large-scale defects can be closed in a single-stage procedure and may be especially required in acute burn reconstruction.

**Figure 2:**
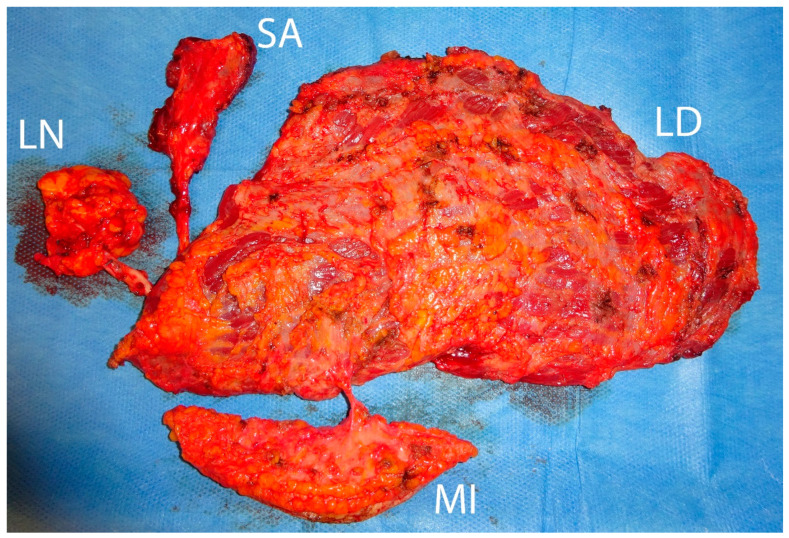
Raised compound flap consisting of a latissimus dorsi muscle flap (LD), a serratus anterior muscle flap (SA), a perforator-based monitor flap (MI), and a thoracodorsal lymph node flap (LN).

**Figure 3:**
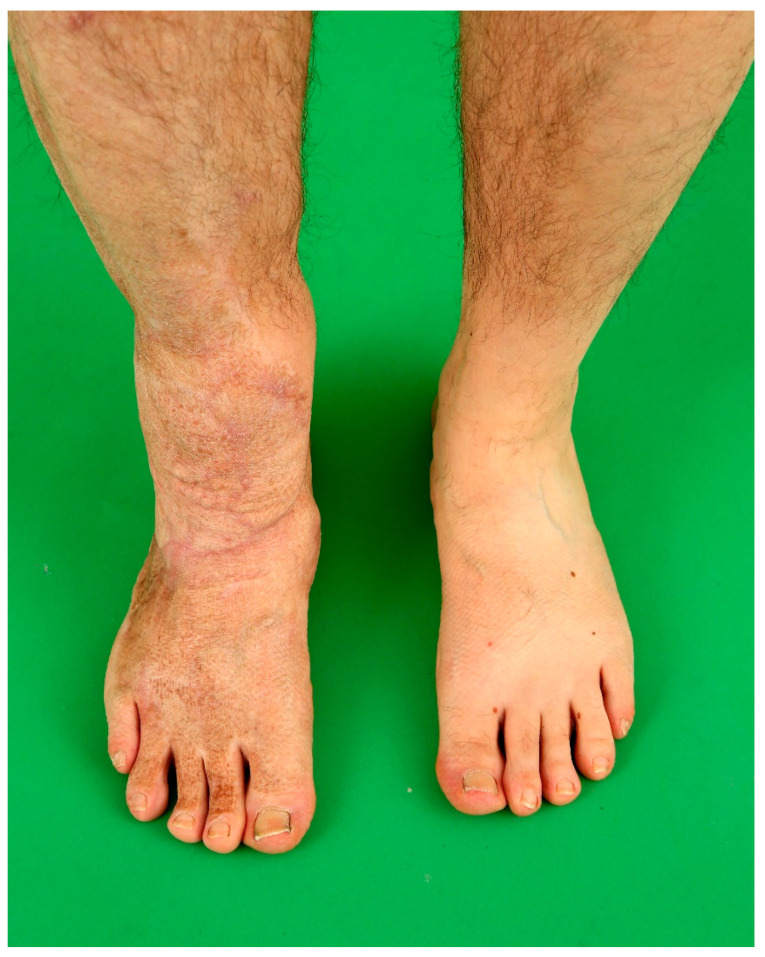
Postoperative outcome one year after free flap procedure. Both muscle flaps atrophied, providing a slim contour of the right ankle. Proper lymph drainage without secondary edema of the right foot is seen.

Severe and circumferential damage to skin of the extremities with fascial excision of the burn may lead to acute posttraumatic lymphedema due to local lymphatic vessel injury and interruption, and can be addressed with advanced microsurgical solutions such as compound flaps together with vascularized lymph node flaps (cp. Figure 2 and Figure 3) [26].

**Figure 4:**
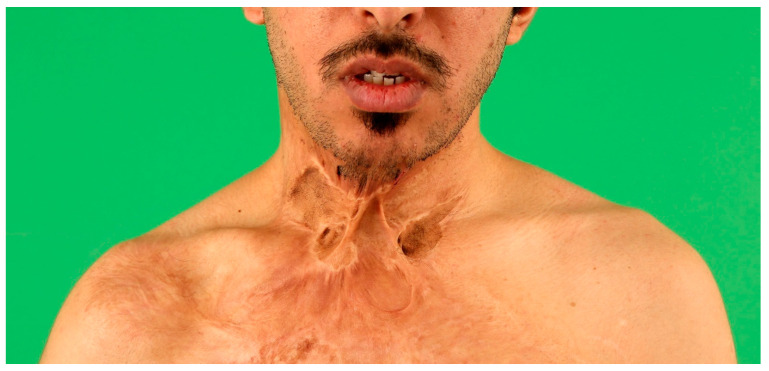
Ventral neck contracture one year following burns resulting from a domestic fire. Reclination of the neck is severely impaired and the patient suffers from pain due to the contracting scar.

**Figure 5:**
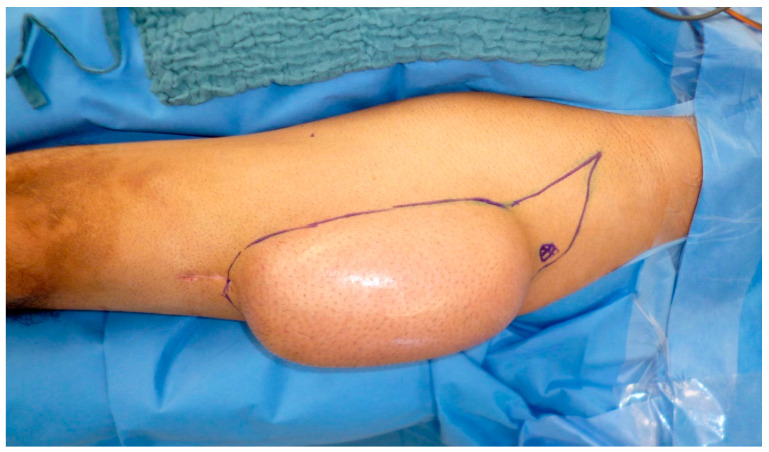
A rectangular expander has been implanted epifascially next to a suitable ALT perforator using intra-operative duplex ultrascan and subsequently filled with 700cc saline solution to pre-expand the planned ALT flap.

**Figure 6:**
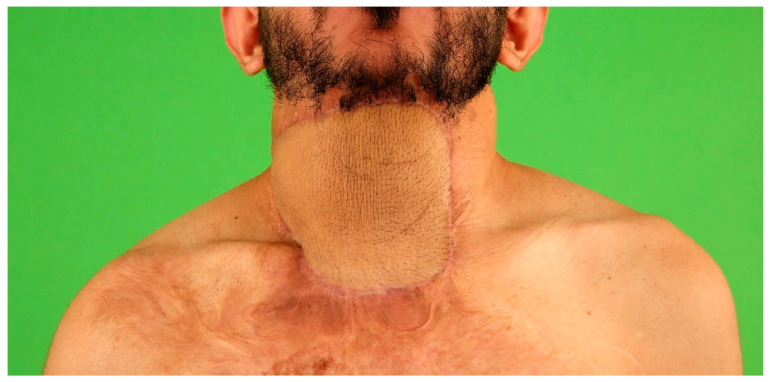
Postoperative result one year after flap transfer. The neck contracture is entirely released, with normal neck movement and pleasant aesthetic outcome.

In cases of secondary burn scar reconstruction where defect closure is not urgent, a two-stage approach with pre-expansion of the planned free flap and secondary flap transfer is possible to allow large-scale replacement of contracture scars [27,28]. Figure 4, Figure 5 and Figure 6 show the reconstruction of a sternomental contracture using a pre-expanded ALT flap.

## 5. Recipient Site

At the recipient site, radical wound debridement prior to flap transfer is necessary to reduce microbial colonization of the wound bed and to provide the best conditions for wound healing and flap integration. Thorough evaluation of the recipient vessels is crucial, especially after electrical trauma or other deep thermal trauma [29] and should include an MRA or CT angiography for the affected lower extremity and also for the upper extremity in patients with high voltage trauma. Since altered recipient vessels tremendously increase the risk of flap failure, arteriovenous loops should be used to approach the recipient vessels in uninjured areas whenever possible [30]. In contrast to defects resulting from other causes, burn wounds requiring free flap closure are often surrounded by scarred or already grafted skin areas. This lack of normal skin and subcutis often necessitates flap coverage of the flap’s pedicle and the anastomosis site to prevent potential vessel compression. In these cases, an appropriate flap design, e.g., using a “Tennis racquet-like” shape should be considered.

## 6. Outcome and Complications

Microvascular free tissue transfer is often considered to bear a high risk of flap failure in patients injured by major burn trauma. Hemodynamic instability, hypercoagulability, and impaired wound healing caused by systemic inflammation and metabolic alterations can lead to an increased complication rate, particularly in primary burn reconstruction [31,32]. Hence, flap loss has been reported in literature in 12–20% of acute free flap transfers [19,33]. Of note, most flap failures were observed in patients suffering from high-voltage injuries [19]. This might be due to local damage to the recipient vessels in terms of partial obliteration, intima damage or subacute inflammation. In cases with actual electrical high voltage current flow through an extremity, these alterations might be located proximal to the actual defect [29] and might not be easily detectable when inspecting a recipient’s vessel during flap transfer. In contrast, Uslu reports eleven successful reconstructions of defects resulting from high-voltage injuries on ankle and foot by anastomosing ALT flaps in the zone of trauma or immediately adjacent thereto [34].

In our own patient collective of microsurgical reconstruction in primary defect reconstruction after burn trauma, we were able to follow up 13 burn patients receiving 14 free flaps to the upper extremity in a period of three years [12]. While the total burned surface area (TBSA) ranged from 1 to 54%, burn injuries were caused by thermal crush injury (*n* = 5, 39%), contact burn (*n* = 3, 23%), electrical injury (*n* = 3, 23%), and explosion and friction burn injury in one patient each (8%).

**Figure 7:**
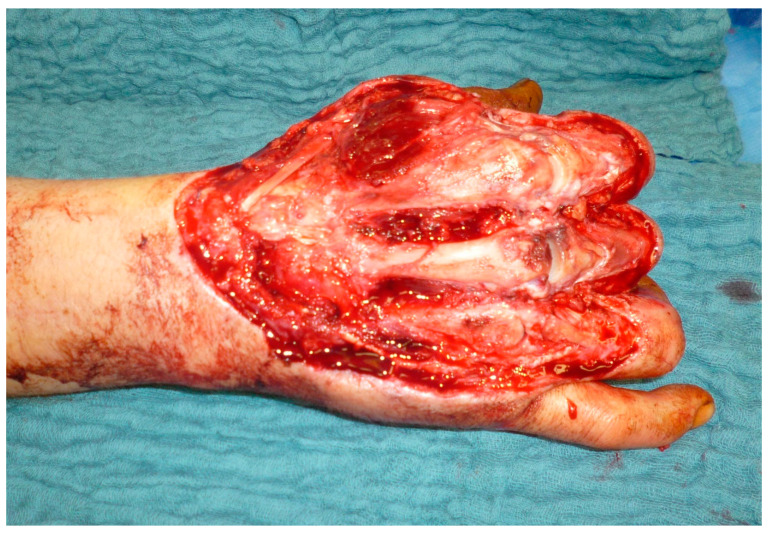
Defect after burn wound debridement of a full-thickness burn to the hand. Necrotic extensor tendons and necrotic capsules of metacarpal joints were resected.

**Figure 8:**
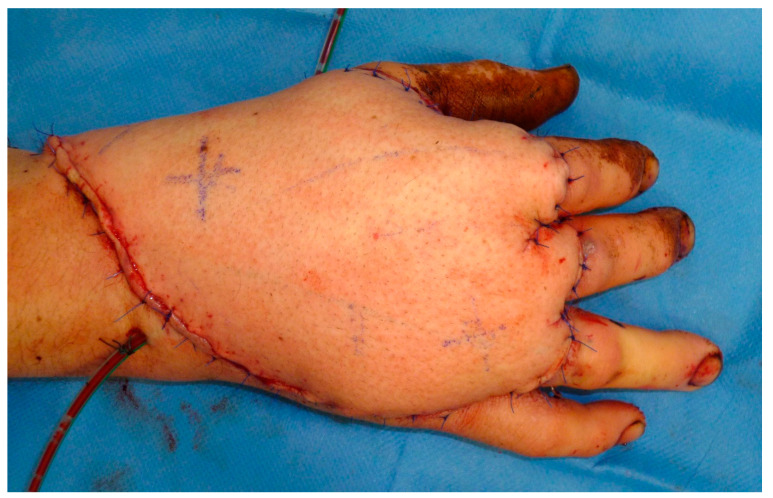
A bipedicular anterolateral thigh flap has been transferred for defect closure after arthrodesis to metacarpal joints 2 and 3 and extensor tendon reconstruction with autologous tendon graft.

**Figure 9:**
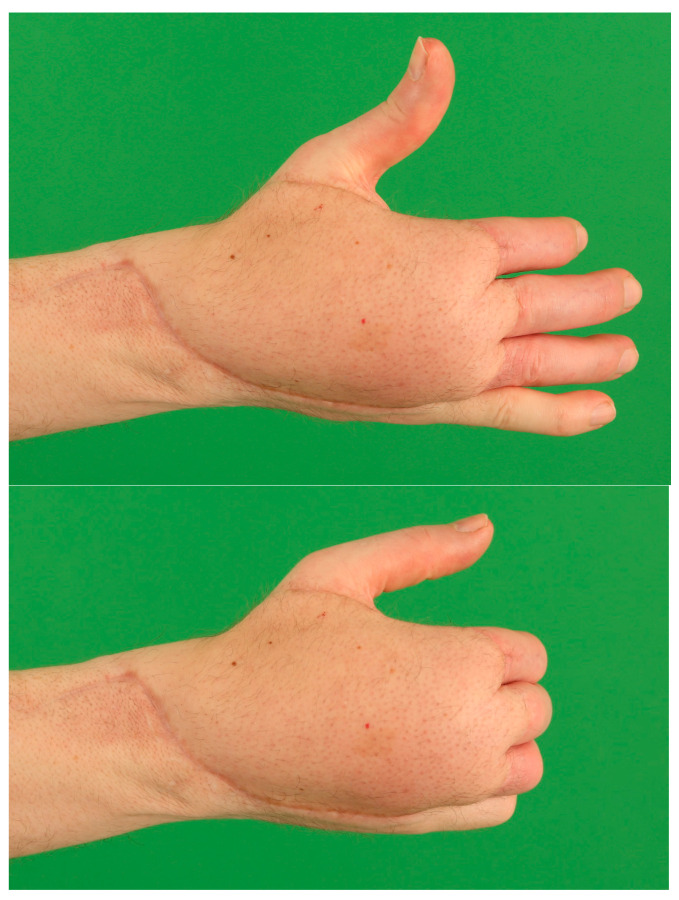
Result six months after free flap procedure. Wound healing is completed and the free flap shows a thin contour facilitating movement of the remaining finger joints.

The ALT was used in most cases (*n* = 9; 64%), followed by latissimus dorsi muscle flaps (LD) (*n* = 3; 18%), serratus anterior muscle flap, and tensor fascia lata (TFL) flap (*n* = 1; 7%). The radial artery was reconstructed using ALT flaps going along with defect reconstruction in two cases. Figure 7, Figure 8 and Figure 9 show an exemplary case of this cohort.

Reconstructive failure due to flap loss was observed in one case (7%), where secondary defect closure using a pedicled groin flap was necessary. In addition, five microsurgical revisions were required because of arterial thrombosis (7%), venous insufficiency (14%), or hematoma (14%). Routine rotational thromboelastometry (ROTEM) during preoperative evaluation showed hypercoagulopathy in ten patients (71%) consistent with a hypercoagulative state that is described as beginning 48 h after trauma [20,35,36]. All documented operative revisions and flap loss occurred in patients with altered coagulation and nearly all complications occurred in patients with a delay of 19 to 30 days between burn trauma and defect reconstruction.

Cho et al. presented a series of 518 free flaps for lower limb reconstruction at the Duke University and the University of Pennsylvania, and showed an overall rate of flap loss of 8.3% [37]. Zhang et al. found a total flap loss rate of 6.0% in a pooled collective of upper limb reconstructions in a recent meta-analysis [38]. Hence, the observed rate of flap failure in our burn cohort is in line with cohorts of other traumatic limb defects undergoing microvascular reconstruction. It shows that free flaps are a reliable tool in defect reconstruction in burned patients when the measures described above were considered. Alteration in coagulation and timing of the free flap procedure seem to be of special relevance in this group of patients.

In secondary scar reconstruction, where the recipient sites are usually closed, non-infected, and hemodynamic and rheological alterations have been compensated, most of the above-mentioned microsurgical risks are absent. Hence, an even lower complication rate has been reported for free flap transfer for secondary burn scar reconstruction, offering a safe and reliable tool in burn scar treatment [27,39].

## 7. Conclusions

Microvascular reconstruction in primary burn reconstruction might have a slightly higher risk compared to free flap reconstruction in general, but from our perspective, these risks and further relative disadvantages like operating time, the need for prompt complication management, and potential donor site defects, are counterbalanced by the numerous advantages—especially the preservation of a significantly injured extremity. Compared to pedicled flaps or two-staged skin grafting, functional training can be started earlier and consequently the functional outcome is favorable. Fewer joint contractures or tendon adhesions, and fewer secondary scar contractures necessitating further surgical procedures are seen. If still needed, secondary procedures like tenolysis or arthrolysis are facilitated after free flap transfer compared to skin grafting or the use of dermal substitutes and can easily be combined with secondary thinning of free flaps. Advanced concepts in acute burn reconstruction include flow-through flaps and vascularized lymph node compound flaps [22,26].

To ensure the best possible outcome in free flap transfer in primary burn reconstruction, recipient vessels must be well-chosen and carefully examined, while arteriovenous loops should be used with a low threshold [30]. To avoid hypercoagulability and chronic inflammation, optimal timing of free flap reconstruction is of key importance for successful microsurgical defect reconstruction.

In burn scar treatment with even smaller risk of free flap failure, microsurgical reconstruction is a cornerstone of burn scar release, offering an effective single-stage reconstructive procedure with a low risk of recurrent scar contracture.
